# Opportunities and challenges for monitoring a recolonizing large herbivore using citizen science

**DOI:** 10.1002/ece3.10484

**Published:** 2023-09-02

**Authors:** Emu‐Felicitas Ostermann‐Miyashita, Hendrik Bluhm, Kornelia Dobiáš, Nina Gandl, Sophia Hibler, Samantha Look, Frank‐Uwe Michler, Leonie Weltgen, Aleksandra Smaga, Hannes J. König, Tobias Kuemmerle, Christian Kiffner

**Affiliations:** ^1^ Faculty of Life Sciences Thaer‐Institute of Agricultural and Horticultural Sciences, Humboldt Universität zu Berlin Berlin Germany; ^2^ Leibniz Centre for Agricultural Landscape Research (ZALF) Müncheberg Germany; ^3^ Geography Department Humboldt‐Universität zu Berlin Berlin Germany; ^4^ Landesbetrieb Forst Brandenburg Abt. 4 Landeskompetenzzentrum Forst Eberswalde (LFE) Eberswalde Germany; ^5^ WWF Deutschland Berlin Germany; ^6^ Faculty of Forest and Environment Eberswalde University for Sustainable Development Eberswalde Germany; ^7^ Zachodniopomorskie Towarzystwo Przyrodnicze Dzika Zagroda Mirosławiec Poland

**Keywords:** *Alces alces*, biodiversity monitoring, elk, human–wildlife coexistence, large‐mammal comeback, participatory research

## Abstract

Monitoring is a prerequisite for evidence‐based wildlife management and conservation planning, yet conventional monitoring approaches are often ineffective for species occurring at low densities. However, some species such as large mammals are often observed by lay people and this information can be leveraged through citizen science monitoring schemes. To ensure that such wildlife monitoring efforts provide robust inferences, assessing the quantity, quality, and potential biases of citizen science data is crucial. For Eurasian moose (*Alces alces*), a species currently recolonizing north‐eastern Germany and occurring in very low numbers, we applied three citizen science tools: a mail/email report system, a smartphone application, and a webpage. Among these monitoring tools, the mail/email report system yielded the greatest number of moose reports in absolute and in standardized (corrected for time effort) terms. The reported moose were predominantly identified as single, adult, male individuals, and reports occurred mostly during late summer. Overlaying citizen science data with independently generated habitat suitability and connectivity maps showed that members of the public detected moose in suitable habitats but not necessarily in movement corridors. Also, moose detections were often recorded near roads, suggestive of spatial bias in the sampling effort. Our results suggest that citizen science‐based data collection can be facilitated by brief, intuitive digital reporting systems. However, inference from the resulting data can be limited due to unquantified and possibly biased sampling effort. To overcome these challenges, we offer specific recommendations such as more structured monitoring efforts involving the public in areas likely to be roamed by moose for improving quantity, quality, and analysis of citizen science‐based data for making robust inferences.

## Graphical abstract

Citizen science‐based monitoring approaches for moose (*Alces alces*) in northeast Germany have been analyzed and their characteristics and limitations have been assessed. We provide concrete technical and structural changes to overcome data collection biases and improve the quantity and quality of citizen science data.

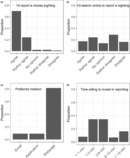

## 1 INTRODUCTION

While the world is facing an accelerating loss of biodiversity and its associated benefits to people (Cepic et al., 2022; IPBES, 2019; IUCN, 2021), some wildlife species are currently making remarkable comebacks. Especially in Europe, several large mammal species have recently experienced major population recoveries (Chapron et al., 2014; Davoli et al., 2022; Linnell et al., 2020), facilitated mostly by strict hunting regulations and protection laws, an expansion of protected areas and other sites acting as wildlife refuges (e.g., former military training grounds), as well as structural changes in agriculture, leading to rural outmigration and land abandonment (Chapron et al., 2014; Churski et al., 2021; Madden, 2008; Navarro & Pereira, 2012; Trouwborst et al., 2015). Understanding the population trends and spatial patterns of large mammals is crucial in areas of their recovery, primarily to proactively assess potential human–wildlife conflicts in the future. Monitoring these animals plays a significant role in this regard.

Eurasian moose (*Alces alces*), a large and charismatic herbivore once widely distributed across Europe, experienced gradual range contraction and local extinctions during the Holocene (Schmölcke & Zachos, 2005). The species survived in Eastern Europe, yet persisted there at low densities during the early and mid‐20th century, due to multiple human pressures, especially hunting, habitat loss, and habitat fragmentation (Niedziałkowska, 2017). In Germany, moose were occasionally reported during the second half of the 20th and beginning of the 21st century. These observations mostly occurred in eastern Germany, in areas close to Poland or the Czech Republic (Schmölcke & Zachos, 2005). In the past, however, moose were frequently shot by hunters or died due to collisions with vehicles (Striese & Heyne, 2021). During recent years, moose observations in eastern and south‐eastern parts of Germany have increased in frequency (Berndt et al., 2021; Janik et al., 2021; Martin, 2013; Schönfeld, 2009; Striese & Heyne, 2021). The implementation of a hunting ban in eastern Germany in 1990, where moose hunting had been permitted during the period of the German Democratic Republic (GDR; 1949–1990), and especially a hunting ban introduced in Poland in 2001 (Borowik et al., 2021), likely contributed to this pattern. In Poland, the moose population has increased drastically since then, and moose are now expanding their distribution range toward the west (Borowik et al., 2018; Raczyński & Ratkiewicz, 2011).

Understanding the distribution of rare species at the edge of their range is relevant for understanding the risks associated with climate change and the adaptation potential of these species (Habibzadeh et al., 2021; Jensen et al., 2020), which could lead to population recovery or habitat expansion. This in turn can inform international and national policy frameworks for species conservation (Robinson et al., 2018) and local management to facilitate sustainable coexistence between humans and wildlife (Lindenmayer & Likens, 2018; Nichols & Williams, 2006). For example, vehicle collisions with moose present a danger for moose and humans alike (Borowik et al., 2021; Jasińska et al., 2019) – a detailed understanding of the spatio‐temporal occurrence of moose is essential to minimize such potential conflicts (König et al., 2020). Effectively monitoring rare species, however, presents formidable challenges (Thompson, 2004). A suite of different monitoring techniques (including direct and indirect sign surveys, GPS‐collaring, camera traps, acoustic‐ and DNA‐based methods) are available to detect and monitor wildlife populations across space and time (Blount et al., 2021; Ford et al., 2009; Kays et al., 2015; Ruppert et al., 2019; Silveira et al., 2003; Sueur & Farina, 2015). However, the implementation of these methods is costly in terms of equipment and human resources (Tarugara et al., 2019). Especially for rare and wide‐ranging species, the cost, time, and effort required to generate sufficient data may be excessive and beyond the budgetary limits of the institutions involved (Shannon et al., 2014). Moreover, even high‐intensity monitoring over long time periods may occasionally fail to detect species known to occur in a specific area (Steinbeiser et al., 2019).

Citizen science, which refers to the voluntary engagement of members of the public regardless of their background in scientific research (Follett & Strezov, 2015), can be a suitable strategy to overcome these challenges. We acknowledge that the term “citizen science” has been criticized for being exclusive (Liebenberg et al., 2021). In light of its widespread adoption, we have chosen to continue using the term “citizen science”. However, when we refer to “citizens,” we intend to encompass all members of the public and use this term inclusively. As the prerequisites strongly differ from traditional scientific research, which is typically constrained by available budgets (Bonney et al., 2014; Commodore et al., 2017; Fontaine et al., 2022), citizen science opens up new possibilities to collect wildlife monitoring data over long time periods and large spatial extents (Dissanayake et al., 2019; Fontaine et al., 2022; Koynova et al., 2021; Ostermann‐Miyashita et al., 2019). Indeed, citizen science data, recorded via a smartphone application, have been successfully used to monitor moose in Canada (Boyce & Corrigan, 2017). However, there are particular challenges when utilizing citizen science‐based data for scientific research, as the search effort of participants is often difficult to quantify, due to less‐structured sampling compared with professional scientific research (Planillo et al., 2021). While studies based on international citizen science platforms have pointed out common temporal, spatial, and taxonomic biases as a result of weakly structured data gathering, these studies have typically focussed on plants, insects, or multitaxa platforms (Binley & Bennett, 2023; Di Cecco et al., 2021; Knape et al., 2022; Meyer et al., 2016; Shirey et al., 2021). Furthermore, it is important to address bias for specific contexts, as factors determining bias likely vary across ecosystems, species, and regions and are especially likely to occur in opportunistic sampling schemes (Geldmann et al., 2016; Johnston et al., 2020).

Effectively adopting citizen science‐based data collection methods to the site‐specific context of moose in Germany requires a nuanced understanding of the technical and social aspects of the monitoring system, as well as the quality of the generated data and how such data can be used for inferring ecological patterns and trends. First, a suite of data collection tools is available, ranging from analog to digital systems, each having its own technical strengths and weaknesses, and user acceptance (Ostermann‐Miyashita et al., 2022; Pateman et al., 2021). Adjusting the means and necessary effort for data reporting to the preferences of members of the public could potentially increase data quantity. In addition, citizen science projects have frequently been criticized for poor data quality due to variable observer skills and sometimes weakly structured protocols (Anhalt‐Depies et al., 2019; Balázs et al., 2021; Binley & Bennett, 2023; Bird et al., 2014; Lewandowski & Specht, 2015). This, however, could be addressed by a more structured project and protocol design, incorporating verification procedures, categorizing data based on clear quality criteria, or applying occupancy models that adjust for observation, detection, and reporting biases (Molinari‐Jobin et al., 2021; Swanson et al., 2016; van Strien et al., 2013; Wiggins et al., 2011). Subsequently restricting data analyses to reliable subsets minimizes possible observer errors, with the trade‐off of data quantity or coverage (Johnston et al., 2021).

Regarding moose in north‐eastern Germany, where the species is currently occurring in low densities and is thought to expand its range, spatial and temporal trends in sightings have been reported, which could be connected to moose behavior. For example, records of registered moose mortalities in north‐eastern Germany (mainly stemming from GDR times) from 1959 to 2020 peaked in September and October. This coincides with the rutting season of moose, suggesting that westward expansion from Poland into Germany might be associated with the rutting behavior (Striese & Heyne, 2021). Provided that reported moose demographics such as age and sex composition of moose are robust, such data could indicate the status of the moose population and inform whether moose use specific areas primarily for explorative movements, dispersal, or for breeding.

To identify opportunities and challenges for effective monitoring of moose in north‐eastern Germany, we addressed the following aspects based on information provided by three independent citizen science data collection tools: a mail/email report system, a smartphone application, and a webpage. We (1) examined the quantity, quality, and characteristics of moose reports based on the three citizen science tools, (2) described the demographic characteristics of the reported moose, (3) analyzed temporal patterns of confirmed moose reports, and (4) assessed associated spatial bias. In addition, we used a survey to (5) gauge the willingness of potential citizen scientists to report moose observations, as well as their preferred medium and time effort for reporting. Based on these results, we provide specific suggestions for increasing the utility of citizen science‐based data for robust moose monitoring in north‐eastern Germany.

## 2 METHODS

### 2.1 Study area

**FIGURE 1:**
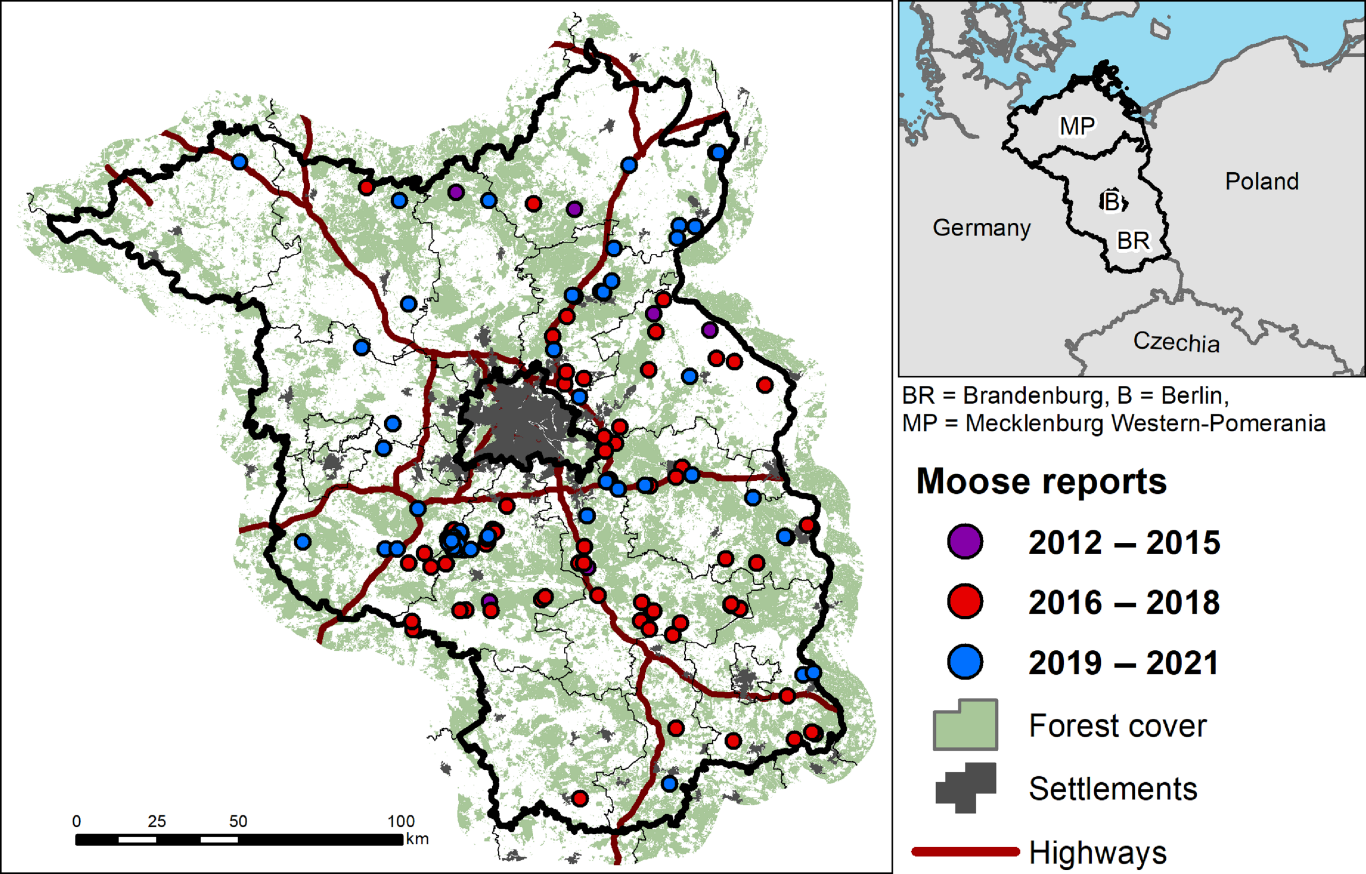
Core study area and locations of all moose reports in Berlin and Brandenburg. The inset in the top right shows the wider study area, highlighting the location of Mecklenburg‐Western Pomerania, Brandenburg, and Berlin in central Europe.

This study was conducted in north‐eastern Germany (Figure 1). The area borders Poland, which supports the largest moose population in central‐eastern Europe (Borowik et al., 2018). Our study spans three federal states of Germany: Berlin, Brandenburg, and Mecklenburg‐Western Pomerania. Overall, the region is characterized by a mosaic of agriculture, managed forests, human settlements, and protected areas. In contrast to most regions in Germany, the human population density in Brandenburg and Mecklenburg‐Western Pomerania is relatively low, standing at 86 and 69 inhabitants per square kilometer, respectively. However, it is essential to note that the city‐state of Berlin stands out with a higher population density of 4127 inhabitants per square kilometer.

### 2.2 Citizen science tools

We used three different citizen science data‐gathering tools in this study: an email/mail system (hereafter mail system), a smartphone application for mammal observations (hereafter application), and a webpage for direct upload of reports (hereafter webpage). The three monitoring tools varied in their spatial coverage of the study area, which we describe in the following sections.

**FIGURE A1:**
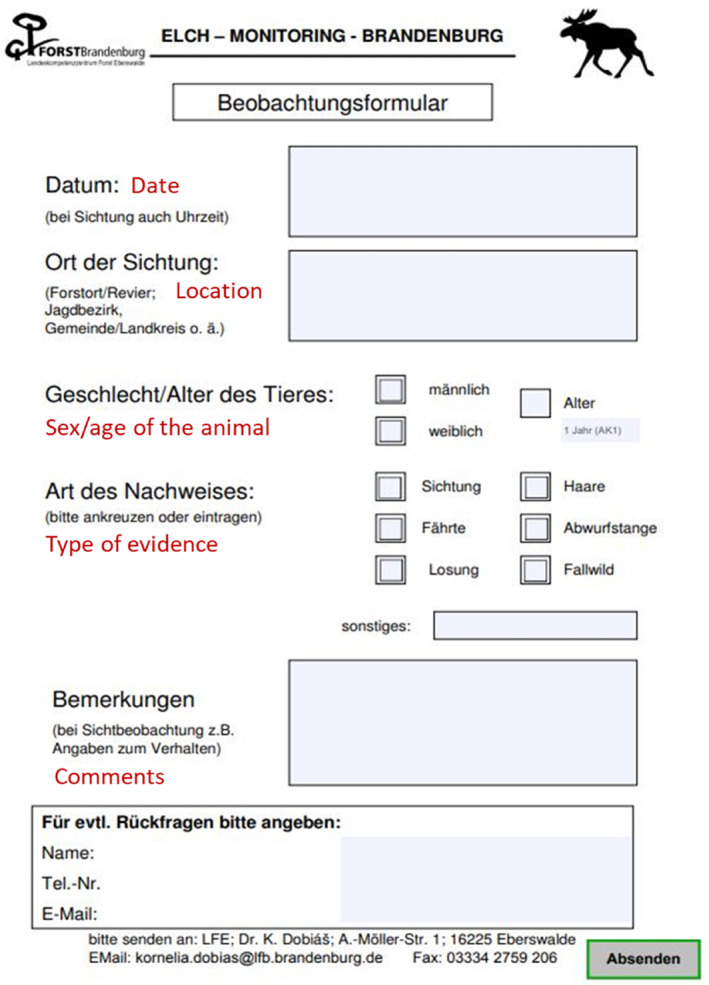
The moose reporting form is on the webpage of the LFE Brandenburg.

The mail system has been in place since 2013. Specifically, the “Landeskompetenzzentrum Forst Eberswalde” (State Forestry Centre of Eberswalde: hereafter LFE) has collected moose occurrence reports from Berlin and Brandenburg State through a form (Figure A1; see https://forst.brandenburg.de/sixcms/media.php/9/elchform.pdf). To report a moose observation, individuals can download a form, and provide details such as the observation date, location and type of observation, and sex and age of the animal. After completing the form, they can then submit it to the LFE either by postal mail or email. Participants are encouraged to attach pictures or videos to the form, but the report can also be processed without such visual evidence. After receiving the completed form with the required contact information, a moose expert will get in touch with the person to verify the report.

The smartphone application *iMammalia* which we considered for this study, was developed as part of the “Mammalnet” project, geared towards mammal species in Europe. Users can upload observations with or without pictures and videos and select the species, location, data, number of individuals, observation method (camera trap, catch, hunted, roadkill, carcass, telemetry, or other), and report type (direct: animal observed alive or dead, and indirect: trail, dropping, den/burrow, or other). After a trial run in four countries (Spain, Germany, Poland, and Croatia), the coverage was extended to additional countries in Europe in 2021 (Blanco‐Aguiar et al., 2022). The application is updated regularly, adding species according to possible range expansions. In this study, we restricted our analyses to records from the German states of Berlin and Brandenburg.

The webpage “www.sichtungmelden.de” was developed as a course project by a group of master students at the Ludwig‐Maximilians Universität München (LMU) in 2021. The website was designed to ease the reporting of moose observations. Participants can choose whether to provide their personal data or not and the necessary reporting information is kept to the minimum with an option to enter the coordinates, time, and to upload a picture as evidence. To make the public aware of the website, six posters were displayed in wildlife parks in eastern Germany. These posters highlighted the features of the project and included a Quick Response (QR) code to direct readers to the website. Once a report was handed in, based on the voluntary contact information, a moose expert then contacted the observer to verify the report.

While the mail system has been active since 2013, the application and the webpage were only implemented more recently. In the *iMammalia* application, moose was added as a target species in late 2020; the website was launched in July 2021. Unfortunately, the planned events to publicize these two tools could not be carried out due to restrictions during the Covid‐19 pandemic. Due to these circumstances, more than 97% of all data were collected through the mail system (Table 1). For these reasons, we restricted all subsequent analyses to the data generated by the mail system dataset. However, we presented all three candidate methods (mail, smartphone application, and website) in the pilot survey explained below, to find out user preferences and analyze the development potential of these tools in the future.

**TABLE 1:** Absolute and relative frequencies of different types of evidence for moose presence in Brandenburg State, Germany, based on 144 reports provided via email/mail from 2013 to 2021.

Type of evidence	Frequency	Percentage
Vehicle collision	6	4.2
Camera trap	8	5.6
Carcass	2	1.4
Sighting	124	86.1
Sighting and tracks	3	2.1
Tracks	1	0.7

### 2.3 Analysis of citizen science data

To analyze the data quality, we used a verification process based on the SCALP criteria. These criteria were initially developed by the project “Status and Conservation of the Alpine Lynx Population (SCALP)” to categorize observation of lynx (*Lynx lynx*) but they are now widely applied to other wildlife species as well (Molinari‐Jobin et al., 2021). The SCALP criteria consider five different categories: C1 records are with direct proof, such as a picture, video, or other hard evidence, including a dead body or genetically identifiable materials (e.g., hair, excrements) of the species. C2 records are confirmed indirect records such as tracks, which have been verified by an expert. C3 records refer to unconfirmed records (i.e., cases in which it was not possible to confirm the record). In instances where records could not be verified due to insufficient information, we categorized them as “NP” (not possible). If lay people clearly misidentified the species (e.g., providing photos of a different species), we assigned the category “False.” Thus, the sum of C1 and C2 reports in comparison with the total number of reports indicates the percentage of the verified results.

To analyze the demographics of reported moose, we evaluated three parameters; group size, sex, and age class (Table 2). For the age category, we considered two classes: adults and juveniles (identified by experts based on pictures). We conducted all statistical analyses in R ver.4.3.0 (R Core Team, 2020). To assess yearly and monthly trends of the confirmed records (i.e., only C1 and C2 records from November 2013 to September 2021), we fitted a Generalized Linear Model (GLM) with Poisson error distribution, to explain the number of confirmed moose reports in a given month as a function of the calendar year (linear predictor) and the month (categorical predictor). We assessed the fit of the model through a rootogram, which is a graphical tool to assess the goodness‐of‐fit of count regression models (Kleiber & Zeileis, 2016). We used the packages “ggplot,” “ggpubr,” “ggsci” for visualization (Kassambara & Mundt, 2020; Wickham, 2016) and “countreg” for the GLM and model diagnostics (Kleiber & Zeileis, 2016).

**TABLE 2:** Distribution of group size, sex, and age classes for moose reported in Brandenburg, Germany from 2013 to 2021. We only considered conformed moose reports (C1 and C2).

	Frequency	Percentage
Group size		
1	83	93.3
2	6	6.7
Sex		
Male	54	56.8
Female	20	21.1
Unidentified	21	22.1
Age		
Adult	93	97.9
Juvenile	2	2.1

### 2.4 Quantifying spatial bias of report locations

We used the spatial information provided alongside each report from the mail system (i.e., coordinates or description of the location) to geo‐reference and map all moose reports in Brandenburg (Figure 1). To assess how moose reports were spatially distributed in relation to known areas of suitable habitats and movement corridors, we used available maps depicting habitat suitability and landscape connectivity for moose (Bluhm et al., 2022). We randomly sampled background points within the study area, equal to the number of confirmed moose reports (i.e., SCALP C1 & C2, *n* = 89), and compared their habitat suitability and connectivity values. Habitat suitability values represented an index ranging from 0 to 1 and were the result of an ensemble of species distribution model (Maxent and Boosted Regression Trees) based on occurrence data from extant moose populations in Europe, and environmental predictor variables (Bluhm et al., 2022). Connectivity values represented the cumulative current density of movement simulations using circuit theory modeling (Dickson et al., 2019; McRae & Shah, 2011), indicating the probability of use for each cell by a moving individual (Bluhm et al., 2022). We expected that moose are more likely to be present in areas of suitable habitat, as well as in areas providing good conditions for movement, and thus hypothesized that the habitat suitability and connectivity values at report locations would be significantly higher than at random locations. However, as citizen science data can have a strong inherent sampling bias, we additionally assessed the proximity to roads, in order to control for a potentially uneven distribution of sampling effort resulting in more records in more easily accessible areas (Di Cecco et al., 2021; Shirey et al., 2021). We did this by calculating and comparing the Euclidean distance to the nearest road (using OpenStreetMap road categories *motorway, trunk, primary*, *secondary*, and *tertiary*) of reports versus random locations. Moreover, we compared the Euclidean distance to the nearest settlement (based on the CORINE land‐cover maps: https://land.copernicus.eu/pan‐european/corine‐land‐cover) and human population density based on the GHS‐POP dataset (Schiavina et al., 2023) between moose reports and random locations. Anticipating a common spatial sampling bias observed in citizen science data‐gathering approaches, we hypothesized that there would be a higher frequency of moose sightings closer to settlements or in densely populated areas (Petersen et al., 2021; Tang et al., 2021). To compare habitat suitability, connectivity, Euclidean distance to the nearest road, Euclidean distance to the nearest settlement, and human population density at moose report locations compared with random locations, we used Mann–Whitney *U* tests (Weiner & Craighead, 2010). For variables with significant differences, we calculated effect size estimates *r* (Cohen, 1988; Fritz et al., 2012); according to Cohen (1988), an *r* value of .1 represents a small effect size, .3 represents a moderate effect size, and values >.5 indicate a large effect size.

### 2.5 Questionnaire on willingness to participate and preferred reporting tool

Prior to the launch of the webpage, we conducted a survey on the motivation and possible challenges for participating in the monitoring of moose. We recruited survey respondents from among LMU students and staff, who were affiliated with the partner institutions of the “ŁośBonasus–Crossing!” project (an EU Interreg project, improving the transboundary wildlife management of European bison and moose in the Polish‐German border region), mostly via direct contact by email. Therefore, the selection of survey respondents was biased toward people with a general interest in ecology and wildlife management. The structured interview contained a question to gauge the overall willingness to participate. Given a scenario of seeing a moose in the wild, respondents were asked if they would initiate an internet search to identify pathways for reporting this observation. Further, the questionnaire contained questions about their preferred means for reporting and about an acceptable duration of the required reporting time. In total, 87 respondents participated in this survey.

## 3 RESULTS

### 3.1 Quantity and characteristics of citizen science data on moose reporting

Due to the low number of reports via the webpage and the application, we restricted further analysis to the reports from the mail system (*n* = 144). The majority of reports from the mail format were visual sightings (86.1%, *n* = 124), followed by camera trap records (5.6%, *n* = 8), vehicle collisions (4.2%, *n* = 6), sightings combined with recorded and documented tracks (2.1%, *n* = 3), carcasses (1.4%, *n* = 2) and one sole record of moose tracks (0.7%) (Table 1). “Camera traps” refer to images recorded on camera traps in the region both by private people as well as those set up for monitoring wildlife crossing infrastructure or the expanding wolf population. Camera trap records were distinct from the “visual sighting” where people took pictures with their phones or cameras. Based on the SCALP criteria, more than 60% (*n* = 89) of all reports were confirmed moose detections, with 75 reports (52.1%) classified as C1 and 14 reports (9.7%) categorized as C2. 53 reports (36.8%) were categorized as C3, and 2 reports (1.4%) could not be classified.

### 3.2 Demography, temporal dynamics, and spatial characteristics of reported moose

Analyzing the demography of reported moose showed that a high proportion of the reports were single, adult, males. Among the confirmed (C1 and C2) reports, participants mostly reported a single moose (93.3%, *n* = 83). Occasionally, reports included detections of two moose (6.7%, *n* = 6). Among the unconfirmed reports, there were few cases of larger moose groups (max. of four individuals). More than half of the confirmed reports were classified as male moose (56.8%, *n* = 54), while females (*n* = 20) and unidentified (*n* = 21) accounted for approx. 20% each. There were two reports of juvenile animals (2.1%) while the vast majority were reported to be adult moose (97.9%, *n* = 93).

**FIGURE 2:**
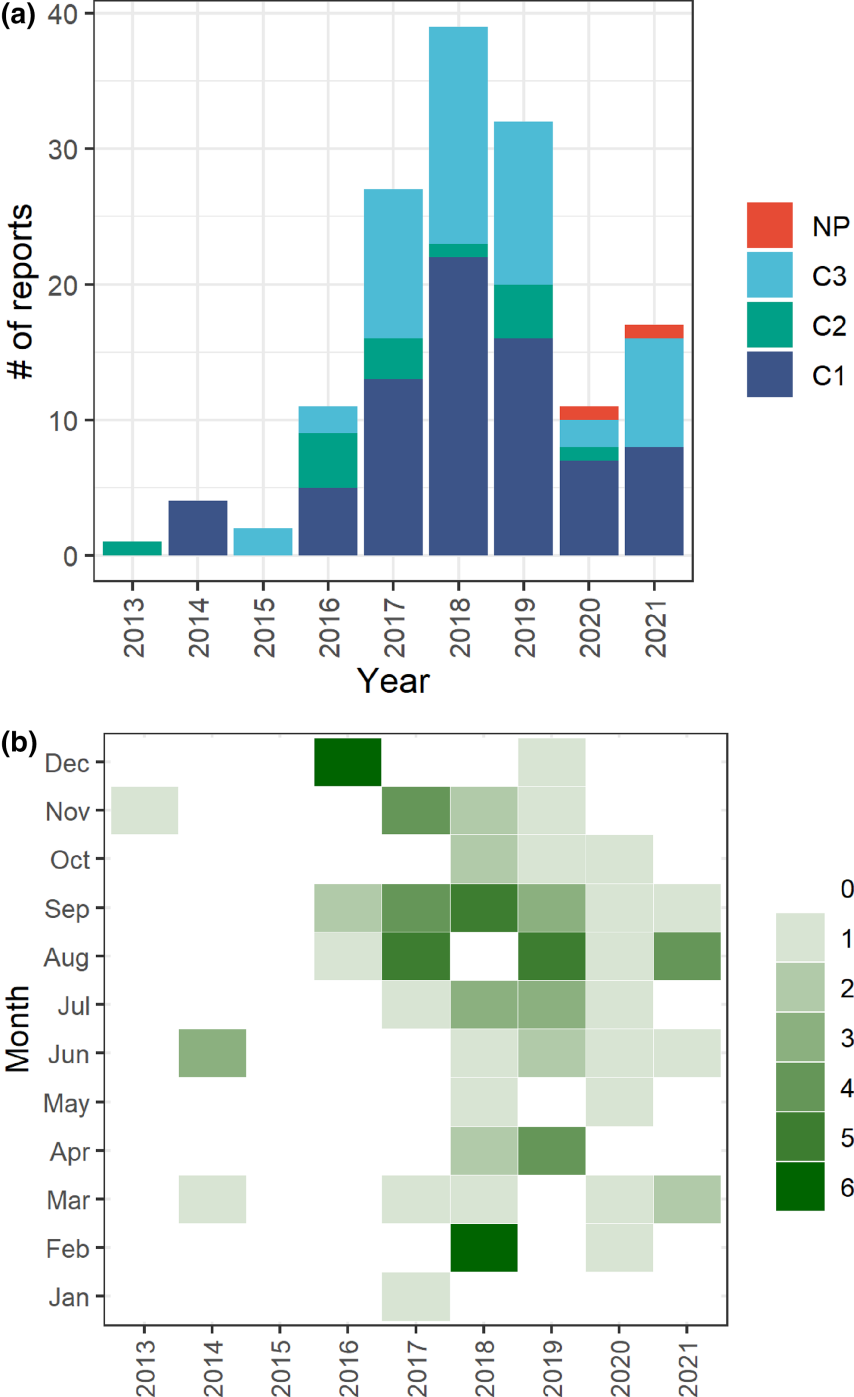
Number of moose reports in Brandenburg State obtained via the email/mail method. (a) The number of reports stratified by scalp criteria and year and (b) the number of C1 and C2 reports in each calendar month from 2013 to 2021.

**FIGURE A2:**
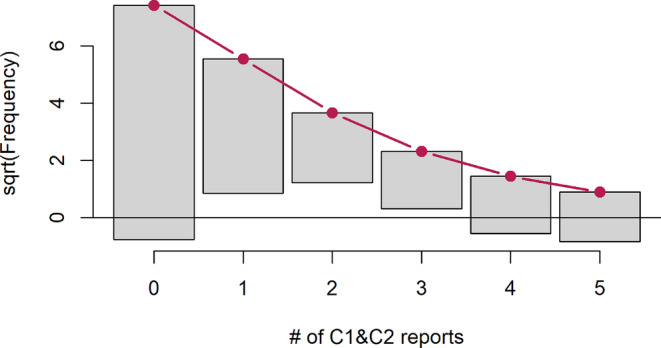
Rootogram of the model to explain temporal (monthly and yearly) variation in reports of moose presence (C1 and C2) in Brandenburg state from 2013 to 2021. Bars indicate observed, and red dots indicate expected (given by the model) values of moose reports. If the bar of the observed values is above the zero line the model overpredicts this particular count bin; if the bar exceeds the zero line, it underpredicts the number of reports.

In terms of temporal dynamics, the number of moose reports has generally shown an increasing yearly trend (regression coefficient *β* of “year” = .164; *p* < .01) since the inception of the mail system in 2013, with a peak in 2018. However, during 2020 and 2021, the number of reports did not reach the frequencies obtained from 2017 to 2019 (Figure 2a). Seasonal trends in moose reports were also evident (Figure 2b). In most years, reports peaked during August and September. The clustering of reports during these 2 months was also corroborated by the significant (for both months *p* < .01) regression coefficients (for both months *β* = 2.773 compared with the reference month January) for these months (Table A1). The rootogram indicates that the GLM model fitted the data relatively well (Figure A2).

**FIGURE 3:**
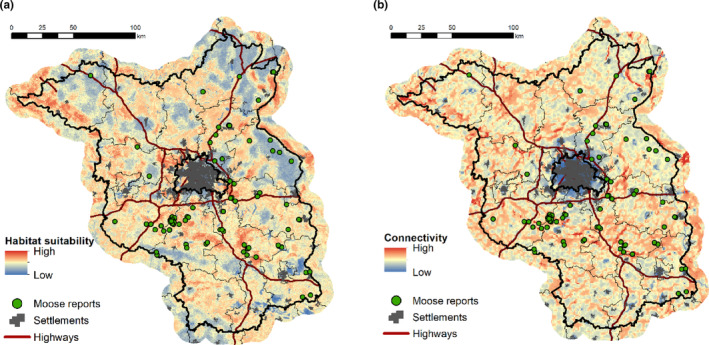
Confirmed moose reports (i.e., SCALP C1 and C2) in Brandenburg plotted over a map of (a) environmental habitat suitability and (b) landscape connectivity for moose. Habitat suitability and connectivity models are detailed in Bluhm et al. (in review).

**FIGURE 4:**
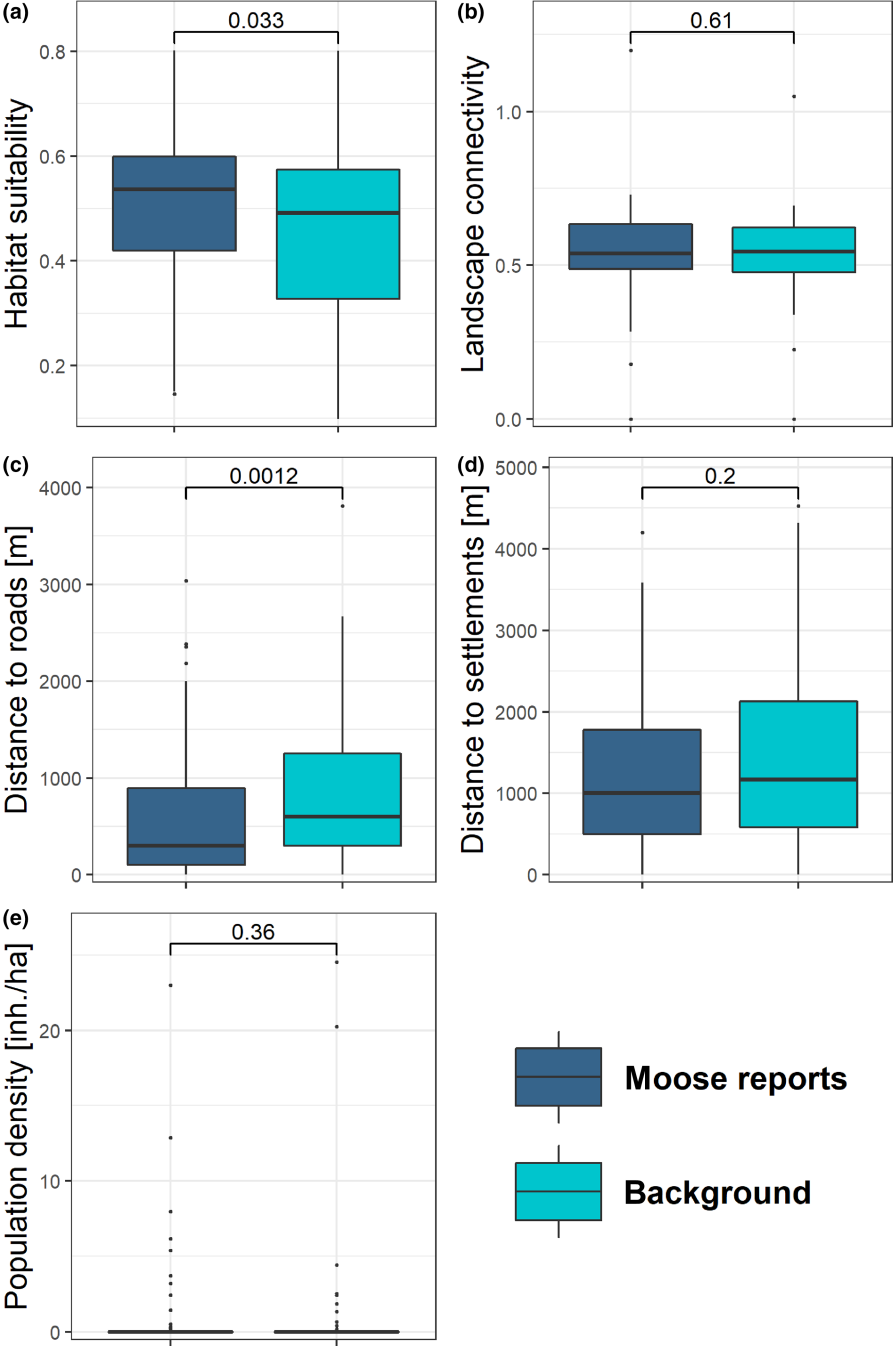
Comparison of the spatial characteristics of the confirmed moose report locations versus randomly sampled background points in Brandenburg. The numbers between the box plots inside each facet represent the *p* values of Mann–Whitney *U* tests.

In terms of spatial characteristics of moose report locations, the majority of moose reports occurred in the eastern part of Brandenburg state, with many reports originating from areas close to the major highways in that region, yet one cluster of reports was also located south‐west of Berlin (Figure 1). Compared with random locations, habitat suitability was significantly (*p* = .033) greater in moose report locations, with a small estimated effect (median habitat suitability index value: random locations = 0.49; moose report locations = 0.54; *r* = .16) (Figures 3a and 4a). In contrast, the locations of moose reports and random locations did not differ significantly in terms of connectivity (Figure 3b and 4b). The assessment of report locations relative to road proximity revealed that the locations of confirmed moose reports were significantly (*p* < .01) closer to roads than random locations (median distance for random locations = 600 m; median distance for moose report locations = 300 m), with a small‐moderate estimated effect size (*r* = .24) (Figure 4c). Distance to nearest settlements (Figure 4d) and the human population density (Figure 4e) did not show significant differences between moose reports and random locations.

### 3.3 Motivation and challenges to engage participants in monitoring

**FIGURE 5:**
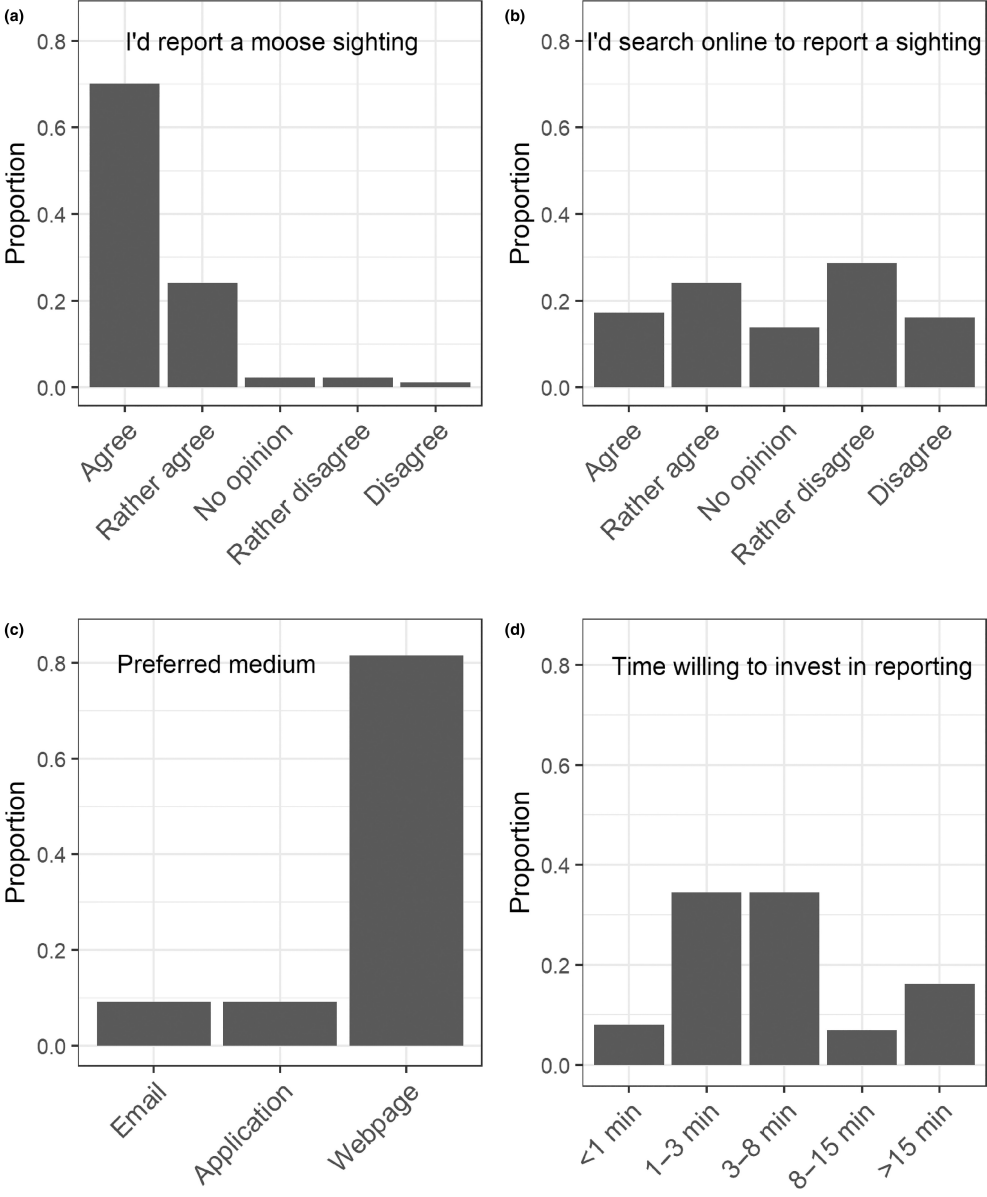
Proportion of responses among students and staff when asked about (a) their general willingness to participate in citizen science‐based moose monitoring, (b) their willingness to actively search online about how to report a moose sighting, (c) their preferred reporting medium, and (d) their preferred amount of time required for reporting.

More than 90% (sum of “agree” and “rather agree”) of the participants answered that they would report a moose if they had encountered evidence of its presence (Figure 5a). However, when asked if they would search for ways to report such evidence on the internet, less than half of the participants answered that they would take this action (Figure 5b). The website emerged as the preferred medium for reporting, accounting for over 80% of the responses, surpassing both email and application alternatives (both under 10%) (Figure 5c). Few participants were willing to invest a maximum of 1 min (8.0%) for the reporting, while a much larger number of respondents (34.5%) indicated that they would prefer the reporting to take less than 3 min. About a third (34.5%) of the participants chose the option “3–8 min,” while 6.9% were willing to invest up to 15 min; 16% of respondents were willing to dedicate more than 15 min (Figure 5d).

## 4 DISCUSSION

Wildlife monitoring is crucial for supporting evidence‐based and sustainable wildlife management, while also fostering human–wildlife coexistence. However, effective monitoring of rare species often faces logistic challenges and financial constraints. Here, we assessed multiple aspects of how members of the public can contribute to monitoring a recolonizing moose population—a first, yet crucial step toward improving moose monitoring in the future. Below, we first discuss demographic, temporal, and spatial aspects of citizen science‐based moose data from north‐eastern Germany, and then discuss how our results can be applied for more effective moose monitoring.

### 4.1 Moose in Germany

Overall, our data showed an increasing trend of moose reports since the beginning of the monitoring program in 2013. However, as the data‐gathering method in this study did not allow for considering differences in on‐the‐ground sampling effort, it is not possible to distinguish whether this reflects an actual increase of moose, an increase of participants in the program, or an increase in the detection probability. Instead of analyzing detailed yearly differences, our focus has been on examining broader temporal patterns and factors that might have influenced the participation of citizen scientists and the abundance of moose. The notable decline of moose records in 2020 and 2021 (Figure 2), coincides with the Covid‐19 pandemic and associated lockdowns and restrictions of human movement in the study area. It is plausible that people were less likely to be outdoors, thus reducing possible survey efforts. In Germany, however, outdoor activities were mostly subject to relatively mild public health measures, especially during the summer months of 2020 and 2021. In other countries, outdoor activities partially increased during the first years of the pandemic compared with prepandemic baselines (Harris et al., 2021; Venter et al., 2020). Similarly, there is little evidence for the possibility that people were less likely to report evidence of moose presence during the pandemic. Evidence from other systems suggests that decreases in human activity had mixed effects on wildlife: while some species expanded their range and increased activity (Vardi et al., 2021), it also hampered the conservation efforts for specific species (Crimmins et al., 2021; Manenti et al., 2020) including monitoring efforts (Stenhouse et al., 2022). As it remains challenging to disentangle the factors underlying the observed yearly trend (i.e., differences in search effort, differences in reporting, or true differences in moose presence), there is a need to develop and implement monitoring methods that are capable to make robust inferences from observations (Pollock et al., 2002). To some extent, this highlights a missed opportunity to assess how the “anthropause” (Gaiser et al., 2022) affected a large herbivore at the western edge of its distribution.

Another possible reason for the recent decline in moose reports is additional movement barriers for the species. Infrastructure development, such as the construction of new highways, is ongoing in western Poland (Ważna et al., 2020). Moreover, fences to prevent the spread of the African swine fever virus have been established along the Polish‐German border (Sauter‐Louis et al., 2022). In Brandenburg, the construction of fences started in December 2020 and was completed in May 2022 with a total length of 255 km (Brandenburg State, 2023); Mecklenburg‐Western Pomerania also constructed fences along sections of its eastern border. The specific type of fence may vary by region, but generally, it comprises two parallel fence lines designed to limit the movement of wild boar and maintain the area between the fence lines free from their presence (https://www.regierung‐mv.de/Landesregierung/lm/Aktuell/?id=172793&processor=processor.sa.pressemitteilung). It is well known that such anthropogenic barriers affect and limit the movement of wildlife (Linnell et al., 2016; Tucker et al., 2018), and reduced connectivity might slow down the westward expansion of moose (Bluhm et al., 2022).

Although yearly variation in moose report frequency may be difficult to explain, the relatively consistent seasonal patterns observed in moose report frequency align well with moose ecology. The seasonal peak of moose reports during the late summer and autumn is likely related to the exploratory movements of young male moose in search of mates during the rutting season (September and October) (Striese & Heyne, 2021). This notion is also supported by the demographic patterns of the observations with a larger number of male moose compared to females. The spatial distribution of moose reports revealed a high number of reports in the eastern part of Brandenburg (Figure 1). This pattern is likely related to the proximity to Poland, where a breeding, and currently increasing population exists (Borowik et al., 2018). The cluster of observations to the southwest of Berlin is located in the Nuthe‐Nieplitz region, the area where a GPS‐collared moose (named “Bert”) established its home range. The high number of sightings in this area might be repeated reports of this individual (Berndt et al., 2021). However, we do acknowledge that our dataset is a detection‐only data set, which does not consider differences in sampling effort.

### 4.2 Benefits and challenges of citizen science data for monitoring moose

In relation to the apparent low number of moose individuals in northeastern Germany, the mail system yielded a substantial quantity of moose records. Furthermore, the high proportion of confirmed records (C1 and C2) also suggests that potential issues of data quality—an argument often raised against citizen science‐based data collection (Lewandowski & Specht, 2015)—can adequately be addressed by applying data quality standards. However, as outlined earlier, the raw number of reports cannot necessarily be used as an index for the population size of moose. Currently, the system is not tailored to identify individuals, thus, the occurrence of double counts cannot be ruled out. In addition, as the sampling effort of people who are willing to report evidence for moose remained unmeasured and likely fluctuated in space and time, the interpretation of raw moose report counts should be approached with caution (Suškevičs et al., 2021).

Similarly, spatial inferences from the moose reports should be interpreted cautiously. In our case study, connectivity values at report locations were not significantly higher than those at random locations, indicating that moose reports did not primarily originate from potential movement corridors. As expected, moose reports originated in areas of high habitat suitability. Nevertheless, it is important to note that the locations of moose reports exhibited a strong association with their proximity to roads. This relationship is likely a reflection of the areas' accessibility and the effort of potential human observers present, rather than indicating an actual preference of moose to be near roads. Distance to the nearest settlement and human population density showed no clear effect. The occasional observations of moose in what appears to be unsuitable habitat (Figures 3a and 4a), also reflect the ability of moose to move through such suboptimal habitat, a behavior that has been observed in other species as well (Farhadinia et al., 2018; Janik et al., 2021; Killeen et al., 2014). Using species occurrence data from citizen science as inputs for modeling habitat selection, habitat suitability, or connectivity can thus be problematic due to the sampling bias inherent to citizen science data (Baker et al., 2022; Johnston et al., 2020).

A key advantage of citizen science is that it enables members of the public to be part of research (Bonney et al., 2009; Turrini et al., 2018), to gain knowledge and insights about the current situation of the target species (Jordan et al., 2011), which can contribute to higher acceptance and more positive attitudes towards the species (Ostermann‐Miyashita et al., 2023). Although the interviewed population in our pilot survey was not representative of the general public, the results showed a high (>90%) willingness to engage in moose monitoring efforts (Figure 5). This might partly reflect the fact that many participants in the survey were students and employees of academic institutions, supporting findings that the young generation (Giachino et al., 2021; Haugestad et al., 2021) and people with more environmental knowledge tend to be more willing to contribute to scientific research (Decker et al., 2010; Haywood et al., 2016). However, evidence from other studies suggests that the interest in participating in wildlife monitoring is fairly high among the general public (Koynova et al., 2021; Stenhouse et al., 2021).

Although the mail system produced the most records in our study, the questionnaire results (Figure 5) indicate a clear preference for a webpage reporting system (>80%). This aligns with the notion that the majority (>70%) of participants did not want to spend more than 8 min for reporting (Figure 5). Specifically for this study, it has to be taken into consideration that the application and the webpage had been active for less than a year compared with the 8 years of the mail system, and the fact that advertising for these newer reporting systems has been hampered by restrictions due to the Covid‐19 pandemic. However, the success of many citizen science‐based wildlife monitoring projects is based on smartphone applications and online tools (Groom et al., 2021), suggesting that developing these digital options and keeping the reporting “short and simple” can attract more participants in the future, possibly leading to increased data quantity.

### 4.3 Toward effective, robust, and long‐term moose monitoring

To fully leverage the potential of citizen science as a valuable raw data source, especially when continued over a longer time span (Bonney et al., 2014; Follett & Strezov, 2015), a number of obstacles need to be overcome. To further improve citizen science data collection protocols and analysis, we consider two different aims for moose monitoring: (1) inferences about the spatial distribution of moose and (2) inferences about population size and demography.

Empirical studies for the movement and distribution of large mammals such as moose have been challenging due to their wide‐ranging and flexible movement patterns (Cushman et al., 2013; Killeen et al., 2014). If the monitoring is primarily targeted to better understand the spatial distribution of the moose population, the apparent spatial bias needs to be taken into account. As the reports exhibited spatial bias and occurred closer to roads than expected, it is not recommendable to naively apply such opportunistic data for habitat modeling of moose in north‐eastern Germany. However, by comparing with other data sources such as movement data of GPS‐collared individuals, by considering additional variables such as search effort (Stenhouse et al., 2020), or by systematically organizing citizen science data collection efforts (Planillo et al., 2021), citizen science data could provide important information about the general distribution of moose. Specific examples would be: considering absence information by comparing with reports of other mammalian species on the iMammalia application or engaging local hunters across large spatial extents in a more structured manner (Månsson et al., 2011). On the other hand, independently obtained information from habitat models can be utilized to maximize the data quantity generated via citizen science methods: targeted communication (e.g., local media, posters) in areas with high habitat suitability, could increase sampling effort in these areas, possibly resulting in greater data quantity. When advertising citizen science projects, it is important to identify the target group (e.g., students, people with specific professions such as hunters, or the general public) (Pateman et al., 2021) and to understand their motivation for participation (Larson et al., 2020). Ensuring the visibility of these projects through public relation activities and providing regular feedback to participants is essential for fostering long‐term contributions (Bíl et al., 2020). Overall, strategic investments (e.g., development of adequate monitoring tools or targeted information campaigns) and coordination are needed for citizen science to reach its full potential as a wildlife monitoring method (Isaac et al., 2014).

When aiming to make inferences about population size or population dynamics, the monitoring approach would ideally consider the implementation of spatially explicit mark‐re‐sight models. These models are extensions of classic capture–mark–recapture models and use spatially explicit information on sighting histories to infer population size. For example, based on unstructured spatial sampling, such models have been implemented effectively for estimating the population sizes of African lions (*Panthera leo*) (Elliot et al., 2020). Such an approach would require sufficiently detailed pictures or videos that allow individual identification of moose. This prerequisite could possibly be met given that a large proportion of moose evidence was based on sightings, often backed up by pictures and opportunistic camera trap pictures (Table 1). Individual identification of moose could further be facilitated by employing modern computer algorithms (Bolger et al., 2012). If conducted over long time periods, such modeling attempts can provide estimates on additional demographic information such as recruitment and mortality as well as movement (Lee & Bond, 2022) and thus provide vital information on the status of the moose population. Alternatively, if individual identification of moose proves too difficult, efforts to calibrate the number of reports with independent and more robust information on population size may provide an alternative avenue to infer changes in population sizes (Månsson et al., 2011).

## 5 CONCLUSION

Overall, our analyses suggest that citizen science approaches are generally suitable for moose monitoring in north‐eastern Germany, yet they also highlight the current limitations of such data. Members of the public not only generated data on the presence of the species but also provided robust data on demography and seasonal occurrence patterns of moose in northeastern Germany. As expected, moose reports occurred predominantly in areas of high habitat suitability but were also associated with proximity to roads—a pattern that likely reflects a bias in search efforts by laypeople. To increase the value of citizen science approaches for long‐term, and large‐scale monitoring of moose populations in Germany and other parts of their distribution (Boyce & Corrigan, 2017; Månsson et al., 2011), we provide multiple recommendations. Increasing the quantity of raw data required for effective moose monitoring could be achieved by establishing a simple and quick reporting system and possibly by publicizing data reporting options in areas with suitable moose habitats and among individuals who frequently spend time outdoors (e.g., hunters, birders, hikers). To make the most of citizen science‐generated data, we emphasize the utility of documenting moose encounters with photographic evidence and the need to account for spatial bias when making inferences from raw observational data. From a more general perspective, our multi‐faceted analysis provides an integrated assessment of the strengths and weaknesses of citizen science data acquisition and offers clear pathways for improving data quantity, quality, and subsequent analyses for making more robust inferences about the fate of the considered wildlife population.

## AUTHOR CONTRIBUTIONS

**Emu‐Felicitas Ostermann‐Miyashita:** Conceptualization (lead); data curation (equal); formal analysis (lead); funding acquisition (equal); investigation (equal); methodology (equal); project administration (equal); resources (equal); visualization (equal); writing – original draft (lead); writing – review and editing (lead). **Hendrik Bluhm:** Conceptualization (supporting); data curation (equal); formal analysis (equal); methodology (equal); resources (equal); visualization (equal); writing – original draft (supporting); writing – review and editing (supporting). **Kornelia Dobiáš:** Data curation (equal); writing – review and editing (supporting). **Nina Gandl:** Data curation (supporting); writing – review and editing (supporting). **Sophia Hibler:** Formal analysis (supporting); investigation (supporting); writing – review and editing (supporting). **Samantha Look:** Writing – review and editing (supporting). **Frank‐Uwe Michler:** Data curation (supporting); validation (equal); writing – review and editing (supporting). **Leonie Weltgen:** Data curation (supporting); writing – review and editing (supporting). **Aleksandra Smaga:** Data curation (supporting); writing – review and editing (supporting). **Hannes J. König:** Funding acquisition (lead); project administration (equal); supervision (equal); writing – review and editing (supporting). **Tobias Kuemmerle:** Data curation (supporting); formal analysis (supporting); funding acquisition (equal); supervision (supporting); writing – original draft (supporting); writing – review and editing (equal). **Christian Kiffner:** Conceptualization (equal); data curation (equal); formal analysis (equal); funding acquisition (equal); methodology (equal); project administration (equal); resources (equal); supervision (lead); validation (equal); visualization (equal); writing – original draft (equal); writing – review and editing (equal).

## FUNDING INFORMATION

This study was financially supported by the Marianne und Dr. Fritz Walter Fischer Foundation, Japanese Student Services Organization (JASSO) and by the EU Interreg project INT144 “LosBonasus – Crossing! Improving transboundary wildlife management for European bison and moose.”

## CONFLICT OF INTEREST STATEMENT

There are no competing interests to be declared for this publication and its research.

## Data Availability

The data that support the findings of this study are available from the corresponding author, E.‐F. Ostermann‐Miyashita, upon reasonable request. The data are not publicly available due to them being highly sensitive location data of a rare species.

## APPENDIX 1

**TABLE A1:** Summary statistics of count regression models with Poisson error distribution, testing the effect of year and month (reference = January) on the number of C1 and C2 reports of moose in Brandenburg state (Germany) from 2013 to 2021.

	Estimate	Std. error	*z*‐Value	*p*‐Value
Intercept	−333.430	87.414	−3.814	<.001
Year	0.164	0.043	3.790	<.001
February	1.946	1.069	1.820	.069
March	1.792	1.080	1.659	.097
April	1.792	1.080	1.659	.097
May	0.693	1.225	0.566	.571
June	2.079	1.061	1.961	.050
July	2.079	1.061	1.961	.050
August	2.773	1.031	2.690	.007
September	2.773	1.031	2.690	.007
October	1.386	1.118	1.240	.215
November	2.079	1.061	1.961	.050
December	1.946	1.069	1.820	.069
